# Caregiving burden, physical activity, psychosocial factors, and maternal cognitive function in mothers of children with developmental disabilities: a cross-sectional study

**DOI:** 10.1186/s40359-026-05259-x

**Published:** 2026-08-03

**Authors:** Ayşe Abit Kocaman, Birol Önal

**Affiliations:** 1 Department of Physiotherapy and Rehabilitation, Faculty of Health Sciences, Kırıkkale University, Kırıkkale, Türkiye; 2 Department of Physiotherapy and Rehabilitation, Faculty of Health Sciences, Atatürk University, Erzurum, Türkiye

**Keywords:** Caregiver, Child with disability, Cognition, Physical activity, Pain

## Abstract

**Background:**

Maternal caregiving for children with developmental disabilities is associated with substantial physical and psychosocial demands that may affect cognitive functioning. This study aimed to investigate factors associated with cognitive function among mothers of children with developmental disabilities using a cross-sectional design.

**Methods:**

This cross-sectional study included 179 mothers of children with developmental disabilities. Sociodemographic characteristics were recorded. Musculoskeletal pain distribution was assessed using the Nordic Musculoskeletal Questionnaire (NMQ), and maternal pain intensity was evaluated with the Visual Analogue Scale (VAS). Cognitive function was assessed using the Montreal Cognitive Assessment (MoCA). Physical activity level, hopelessness, and sleep quality were evaluated using the International Physical Activity Questionnaire–Short Form (IPAQ-SF), Beck Hopelessness Scale (BHS), and Pittsburgh Sleep Quality Index (PSQI), respectively.

**Results:**

MoCA total score was significantly correlated with maternal age (*r* = -0.273), maternal education duration (*r* = 0.513), number of children (*r* = -0.300), child’s age (*r* = -0.341), BHS total score (*r* = -0.581), IPAQ-SF total score (*r* = 0.581), and maternal pain intensity (*r* = -0.357) (all *p* < 0.05), but not with PSQI total score (*r* = 0.080, *p* > 0.05). In hierarchical regression analysis, education duration, physical activity level, hopelessness, and child’s age remained independently associated with maternal cognitive function in the final model (R² = 0.623, *p* < 0.001).

**Conclusion:**

Longer maternal education duration, higher physical activity levels, lower hopelessness, and younger child age were associated with better cognitive function among mothers of children with developmental disabilities. These findings highlight potentially modifiable factors related to maternal cognitive health in this population.

## Introduction

Disability is a broad term referring to impairments, activity limitations, and participation restrictions that may affect an individual’s functioning in daily life [1]. Developmental disorders comprise a group of conditions with onset during the developmental period, including autism spectrum disorder, cerebral palsy, and intellectual disability, among others [2]. Developmental disability is an umbrella term commonly used to describe long-term conditions arising during development that may result in functional limitations in physical, cognitive, behavioral, or adaptive domains. Recent studies have indicated a steady increase in the prevalence of developmental disabilities worldwide [2–4].

Caring for a child with developmental disabilities imposes a substantial physical and emotional burden on parents, particularly mothers, compared with caring for a typically developing child. This burden often limits social participation, weakens support networks, and reduces opportunities for rest and self-care. Consequently, mothers frequently experience anxiety, depression, and high stress, which can impair family relationships and overall quality of life. Additionally, the physically demanding nature of caregiving, such as lifting, transferring, and assisting with daily activities, predisposes these mothers to musculoskeletal problems and chronic pain [5, 6].

Cognitive functioning encompasses multiple domains, including attention, executive functions, memory, language, visuospatial abilities, abstract thinking, reasoning, calculation, and orientation [7]. Attention and concentration involve the ability to focus on relevant stimuli, whereas executive functions refer to higher-order processes such as planning, problem-solving, and cognitive flexibility [8]. Memory includes the encoding, storage, and retrieval of information, and language functions involve both comprehension and expression [9]. Visuospatial skills enable individuals to perceive spatial relationships and navigate their environment, while orientation refers to awareness of time, place, and person [10]. These domains collectively support goal-directed behavior and adaptive functioning in daily life [11, 12]. In caregiving contexts, the integrity of these domains is important for managing daily caregiving tasks, making appropriate decisions, organizing routines, and responding effectively to the child’s needs while ensuring safety. Empirical evidence indicates that maternal executive functions, particularly planning, inhibition, and working memory, are closely related to sensitive caregiving behaviors and may be associated with a lower likelihood of harsh parenting [13]. Additionally, parental reflective functioning, or the capacity to understand the child’s mental states, has been associated with secure attachment and better child emotional outcomes [14]. Maternal decision-making autonomy and perceived safety have also been linked to key caregiving practices, including healthcare seeking, feeding, and safety-related decisions [15, 16]. Such functions may be particularly relevant for mothers of children with disabilities, who often need to cope with continuous physical, emotional, and practical demands in daily life.

Maintaining cognitive health is essential for sustaining daily function and quality of life [17]. Prior research has demonstrated that education, psychological well-being, and physical activity are major determinants of cognitive performance. Individuals with higher educational levels and greater physical activity demonstrate superior executive and memory functions [18, 19]. Psychological factors such as hopelessness have also been identified as factors associated with cognitive decline in later life [20, 21].

Mothers of children with disabilities often experience physical overload due to repetitive lifting and caregiving activities, which may result in chronic musculoskeletal pain, especially in the back, shoulders, and neck [22, 23]. Chronic pain has been associated with alterations in brain structure and function, leading to impairments in attention, memory, and learning [24, 25]. Similarly, sleep disturbances have been shown to negatively influence multiple cognitive domains including language, visuospatial skills, and decision-making [26]. Moreover, previous research suggests that parents of children with disabilities experience faster age-related cognitive decline [27]. Overall, mothers of children with disabilities frequently face pain, sleep problems, hopelessness, and reduced physical activity [22]. Each of these factors has been linked to cognitive dysfunction in various populations [19, 20, 24, 25]. However, to our knowledge, no previous studies have simultaneously examined these variables in this specific population. Therefore, the present study aimed to investigate the relationships between cognitive function and pain, hopelessness, physical activity level, and sleep quality in mothers of children with disabilities.

## Method

This cross-sectional study included mothers of children with developmental disabilities attending a private special education and rehabilitation center in Kırıkkale. Data were collected between January 2023 and January 2024. Participants were recruited using a convenience sampling method from mothers of children receiving services at the center during the study period.

Information about the study’s purpose was provided to the mothers, and face-to-face interviews were conducted for all assessments. Written permissions were obtained from the director of the rehabilitation center and the mothers involved in the study. While demographic information about the mothers was recorded during face-to-face interviews, demographic information about the children was obtained from the mothers. Our study was approved by the Non-Interventional Research Ethics Committee of Kırıkkale University (decision number: 2022.09.15; approval date: 28 September 2022). All procedures were performed in accordance with the Declaration of Helsinki. Written informed consent to participate was obtained from all participants prior to data collection.

Mothers included in the study met the following criteria: having a child diagnosed with a developmental disability (e.g., cerebral palsy, autism spectrum disorder, intellectual disability, Down syndrome, spina bifida, hydrocephalus, spinal cord injury, or brachial plexus injury), being between the ages of 18 and 50 years, serving as the primary caregiver of the child, residing in the same household, having a child aged between 2 and 18 years, exhibiting no communication problems, and reporting pain in at least one area of the musculoskeletal system within the past 12 months. Exclusion criteria encompassed breastfeeding, having a chronic neurological or orthopedic disease, a history of surgery on the musculoskeletal system, and a background of serious or chronic psychosocial disorders before the birth of the children with disabilities. A priori power analysis for multiple linear regression was performed using G*Power 3.1 software. The analysis was based on a medium effect size (f² = 0.15), a significance level of α = 0.05, a statistical power (1–β) of 0.95, and seven predictor variables. According to these parameters, the minimum required sample size was calculated as 153 participants. In the present study, 179 mothers were included, exceeding the required number and providing sufficient statistical power for the regression analyses. A total of 179 mothers of children with developmental disabilities were included in the study. The mean age of the mothers was 38.11 ± 7.79 years. Detailed demographic and clinical characteristics of the participants are presented in Table 1.

**Table 1:** Demographic and clinical characteristics of the sample

	Mean ± SD	Min-Max (Median)	
Mother’s age (years)	38.11 ± 7.79	37 (22–45)	
Number of children	2.27 ± 1.04	2 (1–7)	
Child’s age (years)	9.9 ± 6.51	8 (1.5–18)	
MoCA (0–30)	23.68 ± 4.26	24 (10–30)	
BHS (0–20)	7.69 ± 5.24	6 (0–20)	
IPAQ-SF	1540.34 ± 1572.82	792 (0-6720)	
PSQI (0–21)	5.18 ± 2.36	5 (0–14)	
Pain intensity (0–10)	5.07 ± 1.84	5 (2–8)	
		**n %**	
Marital status	single	73	40.78
	married	83	46.37
	divorcee	9	5.03
	lost her husband	14	7.82
Place of residence	city	156	87.15
	county	19	10.61
	town	1	0.56
	village	3	1.68
Education Level	low literate	4	2.23
	high literate	20	11.17
	primary school graduate	32	17.88
	secondary school graduate	55	30.73
	high school graduate	61	34.08
	postgrauate	7	3.91

Values are presented as mean ± standard deviation or n (%)

*Abbreviations*: *MoCA *Montreal Cognitive Assessment, *BHS *Beck Hopelessness Scale, *IPAQ-SF *International Physical Activity Questionnaire-Short Form, *PSQI *Pittsburgh Sleep Quality Index

### Outcome measures

The present study identified the MoCA score of mothers of children with disabilities as the dependent variable, while the independent variables included maternal age, pain intensity, education duration, hopelessness level, physical activity level, number of children, and the child’s age. All outcome measures in the present study were obtained from the mothers and reflect maternal characteristics.

#### Montreal cognitive assessment (MoCA)

The MoCA is a brief cognitive screening tool developed by Nasreddine et al. [28] to identify mild cognitive impairment. The MoCA consists of 11 items assessing multiple domains of cognitive functioning. Visuospatial and executive abilities are evaluated with a maximum score of 5 points, naming ability with 3 points, attention and concentration with 6 points, language skills with 3 points, abstract thinking with 2 points, delayed recall (memory) with 5 points, and orientation to time and place with 6 points. The sum of these domain scores yields a total score ranging from 0 to 30, with higher scores indicating better overall cognitive performance. Higher scores in each domain indicate better functioning in the corresponding cognitive ability [29]. According to previously proposed interpretative categories, scores of 26–30 are considered normal, scores of 18–25 indicate mild cognitive impairment, scores of 10–17 indicate moderate cognitive impairment, and scores below 10 indicate severe cognitive impairment [28, 30]. In the original scoring procedure, one point is added for individuals with 12 years of education or less. A score of 26 or above is generally considered within the normal range in the original version [28]. The Turkish version of the MoCA has been shown to be valid and reliable for the Turkish population [31]. In the present study, the MoCA demonstrated acceptable internal consistency (Cronbach’s α = 0.79).

#### Beck hopelessness scale (BHS)

The BHS is a 20-item self-report questionnaire developed to assess negative expectations about the future [32]. Each item is answered as true or false and scored as 0 or 1. The total score ranges from 0 to 20, with higher scores indicating greater hopelessness. Scores are commonly interpreted as minimal (0–3), mild [4–8], moderate [9–14], and severe hopelessness [15–20]. The Turkish version of the scale has been shown to be valid and reliable [33]. In the present study, the BHS showed good internal consistency (Cronbach’s α = 0.75).

#### International physical activity questionnaire-short form (IPAQ-SF)

The IPAQ-SF is a self-report questionnaire designed to assess physical activity levels during the previous seven days [34]. Physical activity is expressed in MET-minutes per week, where MET (Metabolic Equivalent of Task) represents the energy cost of physical activities relative to resting metabolic rate. Standard MET values used in the IPAQ-SF scoring protocol are 3.3 METs for walking, 4.0 METs for moderate-intensity activities, and 8.0 METs for vigorous-intensity activities [35]. Total physical activity is calculated by summing the MET-minutes/week across activity categories. According to the IPAQ classification, physical activity levels are categorized as low (< 600 MET-min/week), moderate (600–3000 MET-min/week), and high (> 3000 MET-min/week) [35]. The Turkish version of the IPAQ-SF has demonstrated validity and reliability for the Turkish population [36].

#### Pittsburgh sleep quality index (PSQI)

The PSQI is a self-report questionnaire used to assess sleep quality over the previous month [37]. It consists of 19 self-rated items that generate seven component scores: subjective sleep quality, sleep latency, sleep duration, habitual sleep efficiency, sleep disturbances, use of sleep medication, and daytime dysfunction. Each of these seven domains is scored on a scale ranging from 0 to 3. Each component has a minimum score of 0 and a maximum score of 3, with higher scores indicating greater impairment in the corresponding aspect of sleep. A component score of 0 indicates no difficulty, whereas a score of 3 indicates severe difficulty in that specific sleep domain. These components are summed to yield a global score ranging from 0 to 21, with higher scores indicating poorer sleep quality. A global score greater than 5 is commonly used to identify poor sleep quality [37]. The optional questions completed by a bed or room partner were not used in the present study. The Turkish version of the PSQI has demonstrated validity and reliability for the Turkish population [38]. In the present study, the PSQI demonstrated good internal consistency (Cronbach’s α = 0.81).

#### Nordic musculoskeletal questionnaire (NMQ)

The questionnaire was originally developed by the Nordic Council of Ministers in 1987 and is a standardized instrument used to assess the presence and distribution of musculoskeletal symptoms across nine body regions: neck, shoulder, elbow, wrist/hand, upper back, low back, hip/thigh, knee, and ankle/foot. Participants report whether they experienced pain in these body regions within the past 12 months using dichotomous (yes/no) responses [39]. The NMQ does not generate a total score; rather, it provides descriptive information regarding the presence and distribution of musculoskeletal pain across body regions. In the present study, the questionnaire was used only to identify pain distribution during the previous 12 months. The cross-cultural adaptation of the scale into Turkish was conducted by Kahraman et al. in 2016 [40].

#### Visual analog scale (VAS)

Pain intensity was assessed using the VAS. Participants were instructed to indicate their level of pain experienced over the past week on a scale ranging from 0 to 10, where 0 indicates no pain and 10 indicates unbearable pain [41]. The VAS score was used as the measure of pain intensity in the statistical analyses.

### Statistical analysis

All analyses were performed using SPSS for Mac, Version 26.0 (SPSS Inc., Chicago, IL, USA). Normality of continuous variables was assessed using the Kolmogorov–Smirnov test. Continuous data were presented as mean (SD), and categorical data as number and percentage. Pearson correlation analysis was used to examine bivariate associations between cognitive function and the independent variables. Hierarchical multiple linear regression analysis was performed to investigate the independent associations between maternal cognitive status and the study variables, including maternal age, education duration, hopelessness level, physical activity level, pain intensity, sleep quality, child’s age, and number of children. Variables were entered into the regression model hierarchically based on both conceptual and empirical considerations. In the first block, maternal age, education duration, pain intensity, and hopelessness level were included as proximal individual characteristics that may be associated with maternal cognitive functioning. In the second block, physical activity level was added to evaluate its additional association beyond these baseline individual factors. In the third block, child-related caregiving characteristics (number of children and child’s age) were included to determine whether they explained additional variance in maternal cognitive function. Sleep quality was considered a theoretically relevant variable and was initially evaluated as a candidate variable; however, because it did not show a significant bivariate association with cognitive function in the present sample, it was not retained in the final regression model in order to preserve model parsimony. Before regression analysis, the assumptions of normality and multicollinearity were assessed. Multicollinearity was evaluated using tolerance and variance inflation factor (VIF) values, with tolerance values < 0.20 or VIF values > 5 considered indicative of problematic multicollinearity [42]. In the present study, tolerance values ranged from 0.70 to 0.82 and VIF values ranged from 1.21 to 1.43, indicating no evidence of multicollinearity among the independent variables. R² was used to determine the proportion of variance explained after the entry of each block. A p value < 0.05 was considered statistically significant.

## Results

A total of 179 mothers of children with developmental disabilities were included in the study. The mean age of the mothers was 38.11 ± 7.79 years, and mothers had a mean of 2.27 ± 1.04 children. The mean age of the children was 9.90 ± 6.51 years. Regarding marital status, 46.37% of the mothers were married, 40.78% were single, 7.82% had lost their husbands, and 5.03% were divorced. Most participants lived in urban areas (87.15%), whereas 10.61% resided in counties and only a small proportion lived in towns or villages. High school graduates constituted the largest educational group (34.08%), followed by secondary school graduates (30.73%) and primary school graduates (17.88%). Mean scores were 23.68 ± 4.26 for the MoCA, 7.69 ± 5.24 for the BHS, 1540.34 ± 1572.82 MET-min/week for the IPAQ-SF, 5.18 ± 2.36 for the PSQI, and 5.07 ± 1.84 for pain intensity. Detailed demographic and clinical characteristics of the participants are presented in Table 1.

**Fig. 1:**
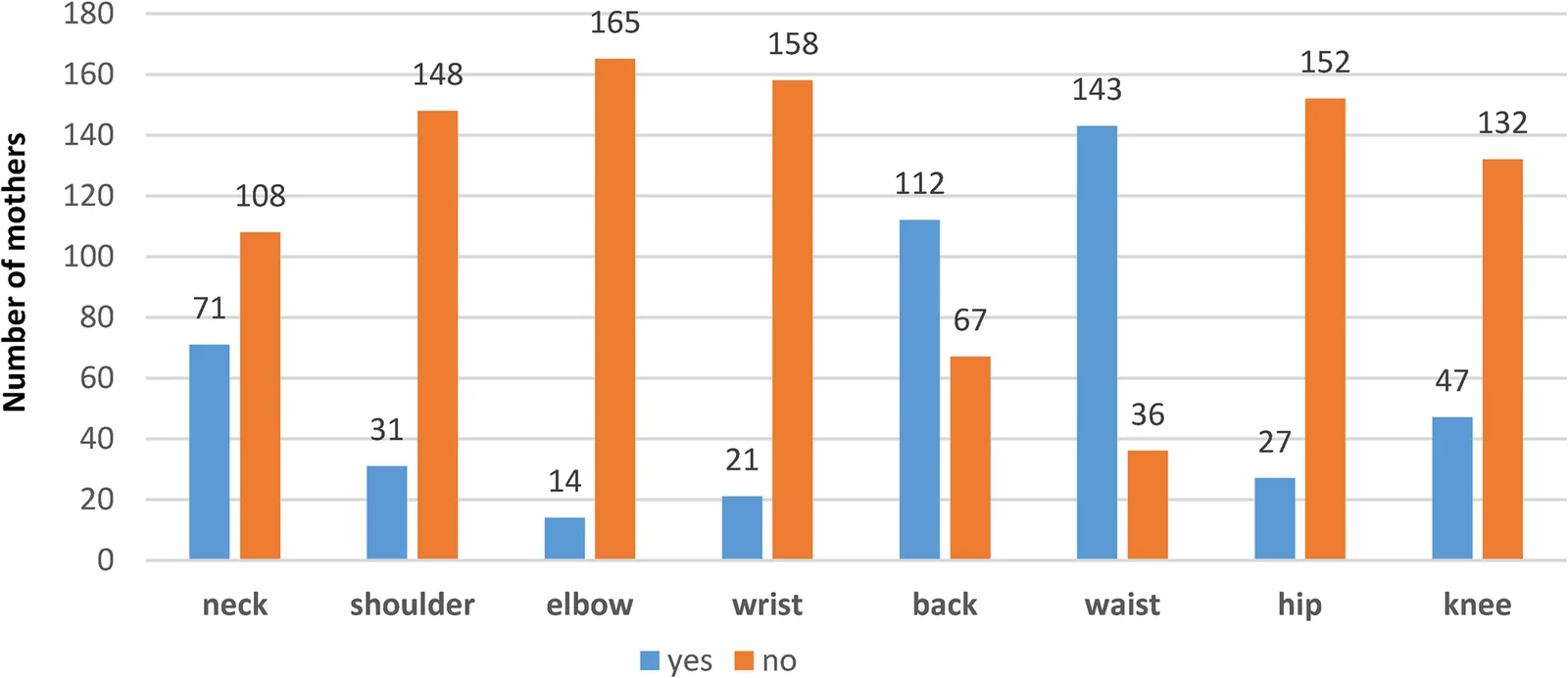
Distribution of musculoskeletal pain among mothers according to body regions (Nordic Musculoskeletal Questionnaire, past 12 months)

**Fig. 2:**
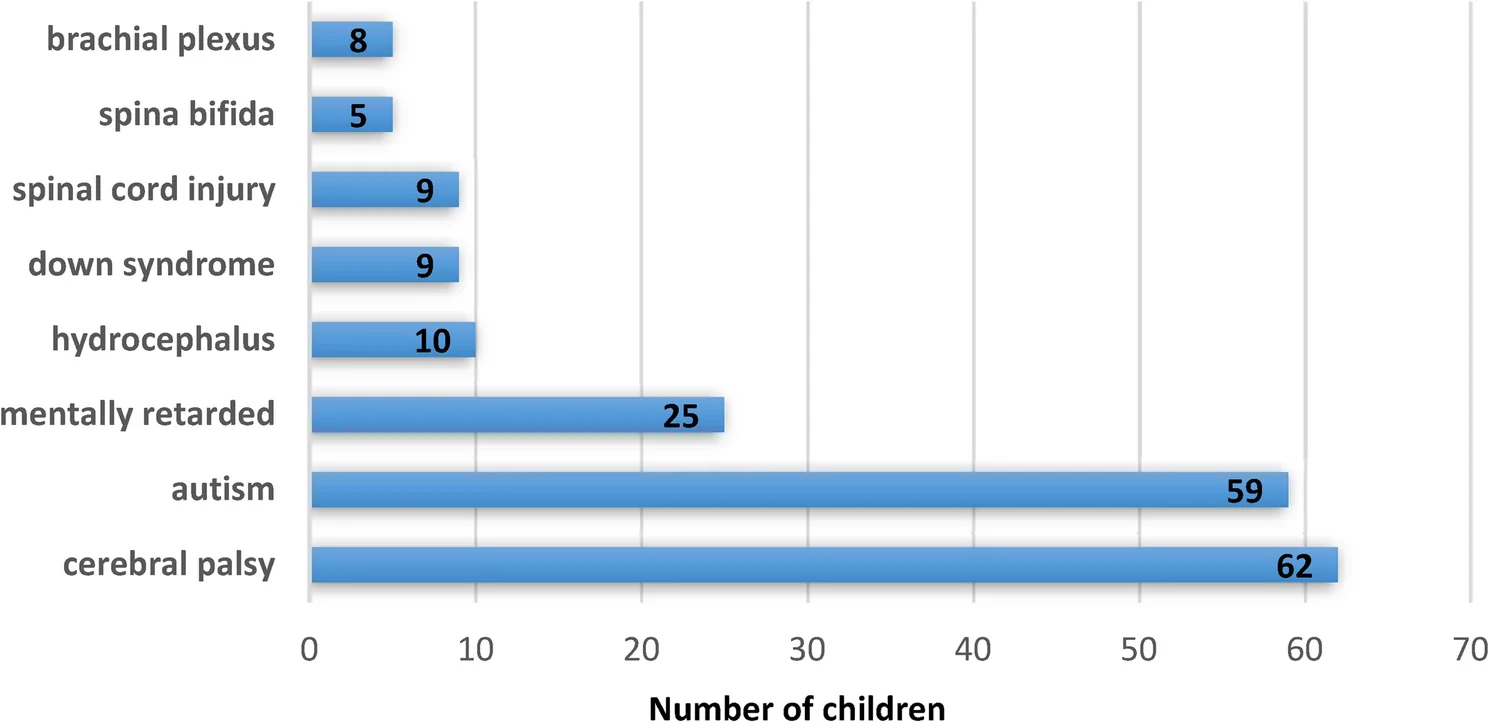
Distribution of disability types among children. Note: Some children had more than one disability; therefore, the total percentage exceeds 100%

The distribution of musculoskeletal pain sites among mothers and the types of developmental disabilities in their children are presented in Figs. 1 and 2, respectively. While various pain locations were reported, the sum of disability types exceeds 100% due to the presence of multiple diagnoses in some children. The results of the univariate correlation analysis are presented in Table 2. MoCA score showed significant positive correlations with education duration (*r* = 0.513, *p* < 0.05) and IPAQ-SF score (*r* = 0.581, *p* < 0.05), whereas significant negative correlations were observed with maternal age (*r* = -0.273, *p* < 0.05), number of children (*r* = -0.300, *p* < 0.05), child’s age (*r* = -0.341, *p* < 0.05), BHS score (*r* = -0.581, *p* < 0.05), and pain intensity (*r* = -0.357, *p* < 0.05). No significant association was found between MoCA and PSQI scores (*r* = 0.080, *p* > 0.05).

**Table 2:** Correlations between independent variables and cognitive function

	MoCA	Mother’s age	Education duration	Number of children	Child’s age	BHS	IPAQ-SF	Pain intensity	PSQI
MoCA	1								
Mother’s age	-0.273*	1							
Education duration	0.513*	-0.334*	1						
Number of children	-0.300*	0.403*	-0.315*	1					
Child’s age	-0.341*	0.273*	-0.137	0.381*	1				
BHS	-0.581*	0.178*	-0.201*	0.179*	0.218*	1			
IPAQ-SF	0.581*	-0.115	0.214*	-0.198*	-0.163*	-0.372*	1		
Pain intensity	-0.357*	0.058	-0.165*	0.178*	0.175*	0.253*	-0.467*	1	
PSQI	0.080	-0.089	0.168*	-0.033	0.106	-0.091	0.026	-0.069	1

Correlation is significant at the 0.05 level (2-tailed)

*Abbreviations*: *MOCA *Montreal Cognitive Assessment, *BHS *Beck Hopelessness Scale, *IPAQ-SF *International Physical Activity Questionnaire-Short Form, *PSQI *Pittsburgh Sleep Quality Index

*p < 0.05 indicates statistical significance

Table 3 shows the results of the hierarchical regression analyses. All models reached statistical significance (Model 1 F = 49.604, *p* < 0.05; Model 2 F = 37.952, *p* < 0.05; Model 3 F = 5.033, *p* < 0.05). In the first model, mother’s age, education duration, pain intensity, and BHS score explained 52.2% of the variance in cognition. For this model, pain intensity (B=-0.414, β =-0.178, *p* = 0.001), education duration (B = 0.423, β = 0.373, *p* < 0.001), and BHS score (B=-0.366, β = -0.451, *p* < 0.001) were significantly associated with cognitive status of mothers of children with disabilities.

Physical activity level was entered into Model 2, while the number of children and the child’s age were added in Model 3. In Model 2, the combination of mother’s age, education duration, pain intensity, BHS score, and IPAQ-SF score accounted for 60.6% of the variance in cognitive function. In this model, education duration, pain intensity, and the BHS score remained independently associated with cognitive function, while the IPAQ-SF score (B = 0.001, β = 0.346, *p* < 0.001) was identified as a significant factor related to cognition in mothers of children with disabilities. Higher IPAQ-SF scores were associated with higher MoCA scores. The variables in Model 3 explained 62.3% of the total variance in cognition. While the child’s age added to Model 3 (B=-0.103, β = -0.158, *p* = 0.002) showed a significant independent association with the MoCA score, the number of children (B = 0.038, β = 0.009, *p* = 0.864) was not significantly associated with the outcome. When comparing the standardized beta coefficients (β) across the final model (Model 3), the strongest independent associations of maternal cognitive function were BHS score (β = -0.342), education duration (β = 0.341), and IPAQ-SF score (β = 0.341), followed by child’s age (β = -0.158). These findings suggest that hopelessness, education level, and physical activity have the strongest associations with cognitive function among mothers of children with disabilities.

**Table 3:** Hierarchical regression analysis of factors associated with maternal cognitive function

Model		B	SE	β	*p*	*R* ^2^	F
1	(Constant)	26.463	1.573			0.522	49.604
	Mother’s age	-0.032	0.030	-0.058	0.294		
	Pain intensity	-0.414	0.125	-0.178	**0.001**		
	Education duration	0.423	0.063	0.373	**< 0.001**		
	BHS	-0.366	0.044	-0.451	**< 0.001**		
2	(Constant)	23.086	1.530			0.606	37.952
	Mother’s age	-0.029	0.028	-0.053	0.295		
	Pain intensity	-0.104	0.124	-0.045	0.403		
	Education duration	0.386	0.058	0.341	**< 0.001**		
	BHS	-0.295	0.042	-0.363	**< 0.001**		
	IPAQ-SF	0.001	0.000	0.346	**< 0.001**		
3	(Constant)	22.995	1.505			0.623	5.033
	Mother’s age	-0.010	0.029	-0.019	0.724		
	Pain intensity	-0.066	0.122	-0.028	0.593		
	Education duration	0.387	0.058	0.341	**< 0.001**		
	BHS	-0.278	0.041	-0.342	**< 0.001**		
	IPAQ-SF	0.001	0.000	0.341	**< 0.001**		
	Number of children	0.038	0.222	0.009	0.864		
	Child’s age	-0.103	0.033	-0.158	**0.002**		

*Abbreviations*: *BHS *Beck Hopelessness Scale, *IPAQ-SF *International Physical Activity Questionnaire-Short Form, *B *unstandardized coefficient, *β *standardized coefficient, *SE *standard error

Values shown in bold are statistically significant (*p*< 0.05)

## Discussion

The present study found that education duration, hopelessness level, child’s age, and physical activity level showed significant independent associations with the cognitive functions of mothers of children with disabilities. In contrast, maternal age, pain intensity, number of children, and sleep quality were not significantly related to cognitive outcomes in the final regression model. These findings highlight the complex associations between psychosocial, educational, and lifestyle factors and maternal cognition in the context of caregiving.

Although previous research has addressed the physical and emotional burden of caregiving, no prior studies have specifically examined factors associated with cognitive performance among mothers of children with disabilities. In the first regression model, pain intensity, education duration, and hopelessness level emerged as independent correlates. The negative association between pain and cognition supports existing evidence linking chronic pain with impairments in attention, memory, and decision-making [24, 25]. It has been suggested that chronic pain may act as a continuous stressor, potentially diverting cognitive resources toward coping processes and thereby interfering with performance on attention-demanding tasks. Moreover, prior literature indicates that persistent pain can be associated with alterations in cortical processing in regions responsible for executive control and emotion regulation, which may provide a theoretical framework for the cognitive inefficiencies observed in caregiving mothers [24, 25].

Education duration was another major factor associated with cognitive functioning. This aligns with the cognitive reserve theory, which posits that education and mentally stimulating experiences enhance the brain’s capacity to tolerate pathology and stress [43]. Individuals with higher education tend to perform better in executive and memory-related tasks [18]. Education is also associated with higher cognitive flexibility and adaptive coping strategies, potentially contributing to the maintenance of cognitive health under chronic stress [44]. In addition, higher educational attainment can provide mothers with greater access to health-related information and social support resources [45]. These resources may reduce stress and promote emotional stability, which in turn help sustain cognitive health [46].

Hopelessness was another variable that demonstrated a significant independent association with cognitive performance. This finding supports the notion that hopelessness, beyond being a symptom of depression, is a strong correlate of adverse health outcomes [47]. Mothers of children with disabilities may experience chronic uncertainty regarding their children’s future and their ability to provide ongoing care, leading to sustained negative expectations [22, 48]. Previous research has shown that feelings of hopelessness in midlife predict later cognitive decline and increase the risk of dementia [20]. Thus, psychosocial interventions aimed at reducing hopelessness could help preserve cognitive and emotional well-being in caregiving mothers.

When physical activity level was introduced into the regression model, it emerged as a stronger independent variable than pain intensity. Regular physical activity is known to promote brain health through multiple mechanisms, including neurogenesis, enhanced synaptic plasticity, and improved cerebrovascular function [18, 19]. Previous research suggests that regular physical activity is associated with structural and functional brain changes, including increased hippocampal volume and improvements in executive functions [49, 50]. Beyond its neurobiological effects, engaging in regular exercise may enhance self-efficacy and perceived control, both of which are associated with better cognitive resilience in stressful caregiving environments [51]. Our findings therefore emphasize that, while previous research has shown that physical activity supports physical and mental health, the present study indicates that it is also a significant positive correlate of cognitive functioning in mothers of children with disabilities.

Interestingly, when physical activity was included in the model, pain intensity lost its statistical significance within the model, suggesting an overlapping relationship between the two variables. Chronic pain is often linked to reduce activity levels, while low physical activity is associated with increased pain sensitivity and fatigue [52]. Thus, promoting physical activity may be beneficial for both chronic pain management and cognitive outcomes in mothers of children with disabilities [19, 52]. Finally, the age of the child was associated with maternal cognition. As children grow older, caregiving tasks and emotional stressors may evolve, which correlates with changes in maternal coping patterns and cognitive functioning. This finding suggests that longitudinal changes in caregiving demands should be considered in future research [27].

Although the final model explained 62.3% of the total variance in MoCA scores, the clinical significance of this finding should be interpreted in light of the MoCA’s role as a screening tool rather than a diagnostic instrument [30]. To provide clinical context, the unstandardized coefficients represent the expected change in MoCA score for each one-unit increase in the predictor. In the final model, a one-unit increase in BHS score was associated with a 0.278-point decrease in MoCA score, while a one-year increase in child’s age was associated with a 0.103-point decrease. Similarly, a one-unit increase in IPAQ-SF score was associated with a 0.001-point increase in MoCA score, and each additional year of education was associated with a 0.387-point increase. Previous research have suggested that the minimal clinically important difference for the MoCA may be approximately 1 to 2 points, whereas larger changes may be needed to exceed measurement variability [53]. In this context, the effect sizes observed for individual predictors may be more reflective of subtle variation in cognitive performance than of overt clinically meaningful impairment. Moreover, MoCA performance is known to be influenced by demographic factors, and ceiling effects in cognitively healthy populations may further complicate the interpretation of small score differences [54, 55]. Therefore, while the present findings indicate statistically significant and potentially meaningful associations at the group level, future studies using more comprehensive neuropsychological assessments may help clarify their clinical relevance.

Taken together, the present findings suggest that maternal cognitive function should be considered within a broader biopsychosocial context in mothers of children with disabilities. One important strength of this study is that cognitive function was examined together with physical, psychosocial, and child-related variables within the same analytical framework. Another strength is the use of validated assessment tools to capture these different dimensions. From a practical perspective, the results indicate that clinicians working with these families may benefit from paying greater attention not only to the child’s rehabilitation needs but also to maternal well-being, particularly physical activity and hopelessness. For mothers, approaches that support regular engagement in daily and recreational physical activity, alongside psychosocial support, may be relevant in the context of cognitive well-being.

### Limitation

Several limitations of this study should be acknowledged. First, physical activity was assessed using a self-report questionnaire rather than objective tools such as accelerometers or pedometers, which may have resulted in over- or underestimation. Second, most participants were from urban areas, which may limit the generalizability of the findings to rural populations. Third, the sample consisted of mothers ranging from young to middle adulthood, while those with chronic diseases were excluded; this relatively homogeneous profile may have reduced variability in cognitive outcomes. In addition, detailed data on the severity of the children’s disabilities and family socioeconomic characteristics were not collected. These factors may have influenced caregiving burden as well as maternal cognitive functioning. Another limitation relates to the use of the MoCA as the primary outcome measure. Although the MoCA is useful for global cognitive screening, it may not be sufficiently sensitive to detect subtle, domain-specific cognitive changes in young or middle-aged adults, and small score differences may not necessarily indicate clinically meaningful impairment. In addition, potential interaction and mediation effects among the study variables were not examined, limiting a more comprehensive understanding of the mechanisms underlying maternal cognitive function. Finally, the cross-sectional design precludes causal inference. Future studies should therefore employ longitudinal or interventional designs, incorporate objective measures of physical activity, stress, and brain function, and include more comprehensive neuropsychological assessments. Collecting standardized data on disability severity, socioeconomic indicators, and diagnostic subgroups, as well as recruiting participants from more diverse socioeconomic and geographic backgrounds, would further strengthen the external validity and clinical interpretability of the findings.

## Conclusion

The regression analysis showed that 62% of the variance in maternal cognitive function was explained by education duration, hopelessness level, child’s age, and physical activity level. Among these variables, physical activity level, education duration, and hopelessness level showed the strongest independent associations with cognitive function in mothers of children with disabilities. Higher physical activity levels were associated with better cognitive functioning; therefore, beyond exercise-based support, strategies that facilitate sustainable lifestyle changes and encourage regular participation in recreational and daily-life physical activities may be relevant in the context of cognitive functioning and overall mental health in this population. Lower hopelessness levels were also associated with better cognitive functioning, suggesting that psychosocial support may be important for maternal well-being and cognitive health. Overall, these findings indicate that maternal cognitive function should be considered within a broader biopsychosocial framework and that supporting maternal mental health and daily functioning may also be relevant for caregiving and child well-being.

## Data Availability

Data may be provided upon reasonable request.

## Abbreviations

NMQ: Nordic Musculoskeletal Questionnaire
VAS: The Visual Analogue Scale
MoCA: Montreal Cognitive Assessment
IPAQ-SF: Physical Activity Questionnaire short form
BHS: Beck Hopelessness Scale
PSQI: Pittsburgh Sleep Quality Index
